# Who Has Mycobacterial Disease? A Cross Sectional Study in Agropastoral Communities in Tanzania

**DOI:** 10.1371/journal.pone.0153711

**Published:** 2016-05-23

**Authors:** Andrew Martin Kilale, Esther Ngadaya, Julius Muhumuza, Gibson Benard Kagaruki, Yakobo Leonard Lema, Bernard James Ngowi, Sayoki Godfrey Mfinanga, Sven Gudmund Hinderaker

**Affiliations:** 1 National Institute for Medical Research, Muhimbili Centre, P.O. Box 3436, Dar es Salaam, Tanzania; 2 National Institute for Medical Research, Tukuyu Centre, P.O. Box 538, Tukuyu, Tanzania; 3 University of Bergen, Centre for International Health (CIH), Postbox 7804, N-5020 Bergen, Norway; Fundació Institut d’Investigació en Ciències de la Salut Germans Trias i Pujol, Universitat Autònoma de Barcelona, SPAIN

## Abstract

**Objective:**

To determine and describe clinical symptoms, demographic characteristics and environmental exposures as determinants of pulmonary mycobacterial diseases among patients examined for tuberculosis in agropastoral communities in Northern Tanzania.

**Methods:**

This was a cross sectional study. Sputum samples were collected from patients attending three hospitals in Tanzania, and were investigated for pulmonary tuberculosis by microscopy between November 2010 and June 2012. The patients were interviewed about background information, and potential exposure to mycobacteria.

**Results:**

We examined 1,711 presumptive tuberculosis cases where 936 (54.2%) were males and 775 (45.3%) females. Of all the study participants, 277 (16%) were found to have sputum samples positive for mycobacteria; 228 (13%) were smear positive, 123 (7%) were culture positive and 74 (4%) were positive by both smear microscopy and culture. Of the 123 mycobacterial culture positive, 15 (12.2%) had non-tuberculous mycobacteria. Males were more likely than females to be positive for mycobacteria. Factors associated with mycobacterial disease were loss of appetite, age groups below 41 years, and being a male. Among HIV negative patients, loss of appetite, age below 20 years and being a male were associated with being mycobacterial positive. Among HIV positive patients, males and those patients with a persistently coughing family member were more likely to harbor mycobacteria.

**Conclusion:**

The findings in this study show that both *M*. *tuberculosis* and non-tuberculous mycobacterial strains were prevalent in the study community. Some risk factors were identified. Although the reported predictors may improve screening for mycobacterial diseases, their use requires some precaution.

## Introduction

Mycobacteria are important acid-fast pathogens ranging from obligate intracellular parasites to environmental species [**1**]. Some mycobacteria are saprophytes and others are obligate parasites, most of them are found in soil and water in a free-living form or in diseased tissue of animals. Diseases caused by mycobacteria and the role of the environment as a reservoir of infections to human is well documented [**2**,**3**]. In communities where livestock, wildlife and humans share the same environment, there is opportunity for close interaction and increased potential risk of mycobacterial infection [**4**]. Mycobacterial diseases cause considerable morbidity and mortality in patients with human immunodeficiency virus (HIV) infection [**5**,**6**]. There is evidence that HIV is a major risk factor for clinical tuberculosis as well as for illnesses associated with certain opportunistic non-tuberculosis mycobacteria, such as *Mycobacterium avium-intracellulare* [**7]**. In addition to altering the risk of diseases caused by mycobacteria, the clinical characteristics of tuberculosis in HIV-infected individuals produce a more disseminated infection [**8]** and are more likely to be sputum-negative than persons without HIV [**9**].

Despite reports of existence of other mycobacterial infections in areas known to have high human-environment-livestock/wildlife interaction, available diagnosis is mainly for pulmonary tuberculosis. This is due to difficulties in diagnosing mycobacterial diseases as the clinical manifestation of most of mycobacterial lung diseases are often similar to those of many other diseases. Lack of a reliable, rapid, and inexpensive diagnostic tests to distinguish the pulmonary mycobacterial diseases remains a major obstacle to effective control of tuberculosis in sub-Saharan Africa where tuberculosis and HIV co-infection is common [10]. Sputum smear microscopy, the standard diagnostic test for pulmonary tuberculosis in low-income countries, fails to diagnose a large proportion of the patients [10,11]. Some earlier studies reported on how well clinical signs and symptoms can predict pulmonary mycobacterial diseases [12**–**14]. In HIV-infected adults with unexplained cough and negative sputum smears, the World Health Organization guidelines recommend clinical judgment and chest radiography for diagnosing tuberculosis.

To our knowledge, there is sufficient documentation of studies that have attempted to assess the diagnostic performance of clinical signs and symptoms [10,15]. Specifically, we aimed to describe the following among patients examined for pulmonary tuberculosis in pastoral communities in Northern Tanzania: 1) the demographic characteristics, 2) the associations between determinants and mycobacterial disease, and 3) the association between determinants and mycobacterial disease by HIV status.

## Methods

### Study design

This was a cross-sectional hospital-based study to assess risk factors for mycobacterial disease among hospital patients.

### Study area and population

We enrolled presumptive tuberculosis patients who attended the Haydom Lutheran Hospital in Mbulu district of Manyara region, the Enduleni Catholic Hospital in Ngorongoro district of Arusha region and the Mount Meru regional Hospital located in Arusha Municipal in northern Tanzania. We selected a study area known for its pastoral communities, and we selected three hospitals where we had previous experience in studying mycobacterial diseases and with a substantial number of patients examined for tuberculosis. We selected both government and private-not-for-profit. The study participants presented at the tuberculosis clinic for investigation. The participants had a reason or symptom that caused the clinician to refer them for investigation for tuberculosis, such as persistent cough for two weeks or more, loss of appetite, weight loss, evening fever, and hemoptysis.

For the purpose of this study, pastoralism refers to communities with farmers who grow crops and or keep livestock searching pastures and water. A presumptive tuberculosis patient (formerly “suspect”) refers to an individual presenting to the health facility and being investigated for tuberculosis. In our study a tuberculosis case is an individual bacteriologically confirmed by smear microscopy or culture of a sputum sample. Literacy is the ability to read and write and speak Swahili (the national language). Education level is the highest grade of education that an individual has completed. Semi-urban means settings with business and employment as well as farming activities.

### Data collection

We collected two sputum samples (spot and morning) from all consenting study participants. A specimen taken on the spot was used for routine examination at the hospital for immediate follow-up treatment, and the rest of the samples along with the morning sputum sample were transported to the Central Tuberculosis Reference Laboratory (CTRL) in Dar es Salaam. Sputum samples collected at Enduleni Catholic and Haydom Lutheran Hospitals were packed and transported to Mt. Meru Regional Hospital in Arusha on the same day of collection. Together with the samples collected at Mt. Meru Regional Hospital, the samples from Enduleni Catholic and Haydom Lutheran Hospitals were transported to the CTRL in Dar es Salaam on the second day from the day of collection. Cool boxes packed with ice cubes were used to maintain the temperature of the samples during transportation. Transport of the samples was done using public buses. In Dar es Salaam, the samples were send to the CTRL on the same day of arrival. Collection of sputum samples and processing for culture was done according to the national tuberculosis guidelines [16].

All study participants were interviewed about their demographic background and symptoms related to their illness, and about risk factors for mycobacteria especially tuberculosis. Interviews were conducted using a structured questionnaire. The interview was in Swahili, and the research assistants filled the questionnaire in English. Data collection was conducted from November 2010 to June 2013. Presence of mycobacteria was bacteriologically confirmed either by microscopy or culture.

### Laboratory procedures

The sputum smears were stained using the Ziehl-Neelsen technique. Only the early morning specimen was used for culture because it was the most likely to grow mycobacteria, and it was least likely to be contaminated with other bacteria [17]. After decontamination and digestion of sputum samples with 4% sodium hydroxide (NaOH), a sterile phosphate buffer pH 6.8 was added to neutralize the effect of NaOH. The samples were concentrated by centrifugation at 3000g for 15 minutes. Supernatant was discarded and sediment was re-suspended in small amount (1–2 ml) of phosphate buffer and inoculated on the slants of solid Lowenstein Jensen (LJ) medium. Culture was considered positive if it grew any visible colonies. Samples that failed to show any growth after eight weeks of LJ incubation were classified as negative. Oxygen Preference Test and Twin 80 Test was further carried out. Species identification was done using polymerase chain reaction (PCR). Growth on LJ media containing para-nitrobenzoic acid (PNB 500μg/ml) was considered as non-tuberculous mycobacteria. Participant HIV status was obtained from clinical records of the three hospitals.

### Ethical considerations

This study was approved by theNational Health Research Ethics Review Committee (NatREC) of the Medical Research Coordination Committee (MRCC) at the National Institute for Medical Research (NIMR) in Tanzania prior to its implementation (Approval Reference number: NIMR/HQ/R.8a/Vol. IX/1009). Furthermore, permission was sought and granted by regional, district and health facility authorities as required. Patients obtained information about the purpose, risks, benefits and comfort of the study participants either by reading or having the consent form read to them by the research assistants. All consenting patients signed the consent form prior to interviews and collection of sputum samples. The participants were free to decline interview at any point in time. Research assistants were trained on all important issues before commencement of data collection.

### Data management

Data were double entered, validated and cleaned using EpiData version 3.1 (Epidata Association, Odense, Denmark) and STATA version 11 (STATA Corp Inc., TX, USA) for cleaning and analysis. The Pearson Chi square test was used to compare proportions between the groups. We considered p<0.05 as statistically significant. Multiple logistic regressions were used for assessing determinants of mycobacterial disease. Crude and adjusted odds ratios (OR) with 95% confidence intervals (CI) were reported. Variables giving p≤0.2 in the univariate analysis were included as adjustment factors in the final multivariable regression model, and included sex, age, education, residence, agro-pastoralist, coughing family member, and smoking. Missing values were excluded from the regression models; the highest number of missing values was for the variable ‘age group’, where 6 values out of 159 (4%) were missing among HIV positives and 30 values missing among 505 (6%) HIV negatives.

## Results

### Demographic characteristics

**Fig 1:**
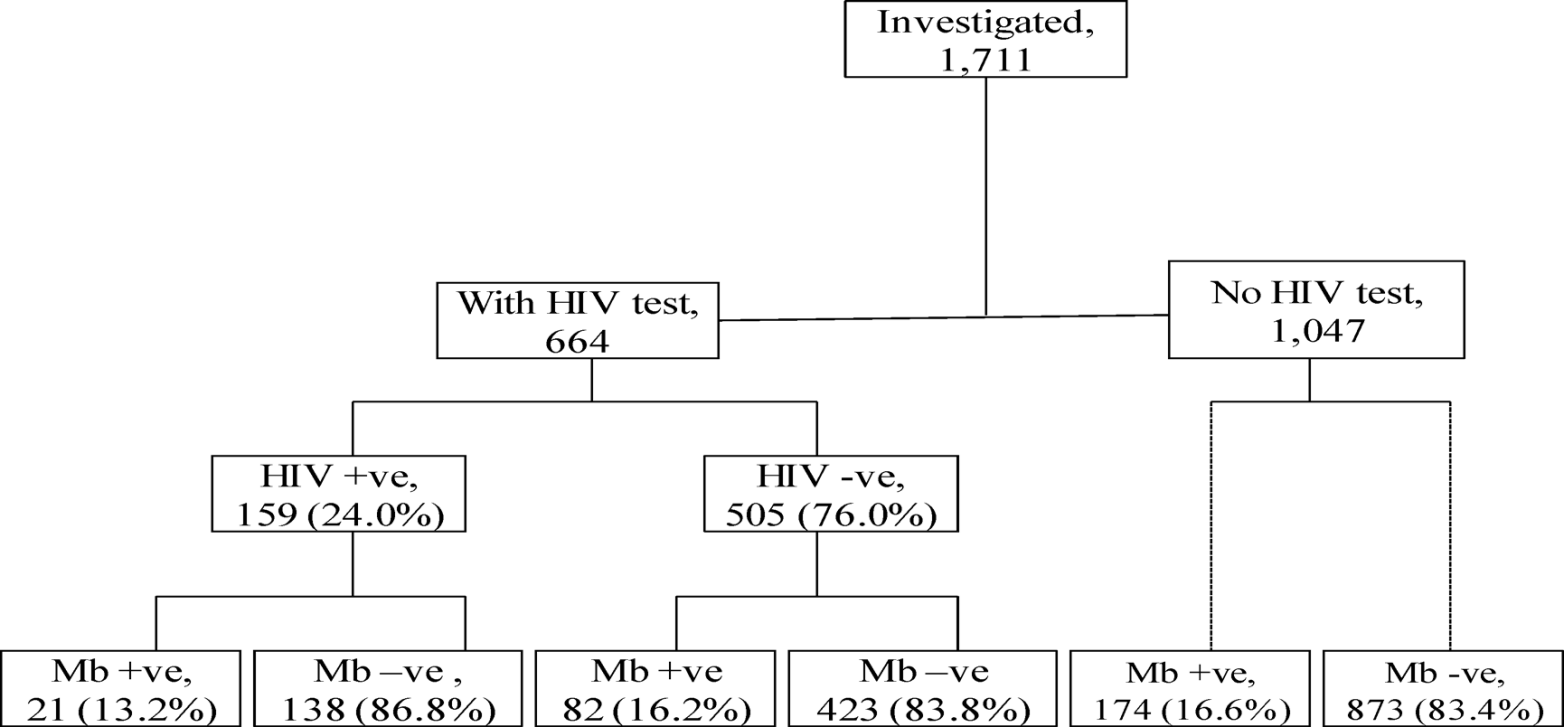
Flowchart of investigations for mycobacteria among study participants, by HIV status, in Northern Tanzania, 2010–12.

A total of 1711 individuals were examined for tuberculosis and the socio-demographic characteristics of the study population are summarized and presented in **Table 1.** Of all the participants, 729 (42.6%) were from semi-urban and 979 (57.2%) were from rural areas. The mean age in years and the standard deviation (SD) of the study participants was 46 (20) for males and 44(20) for females. HIV test results were present in 664 participants of whom 159 (24%) were HIV positive (**Fig 1**).

**Table 1:** Demographic characteristics of 1711 patients examined for tuberculosis in three hospitals of Northern Tanzania, 2010–12.

Demographic characteristic		Tuberculosis suspects examined	
		n	%
Total		1711	100.0
**Sex**			
	Male	927	54.2
	Female	775	45.3
	Missing	9	0.5
**Age group**			
	≤20	148	8.6
	21–30	276	16.1
	31–40	365	21.3
	41–50	261	15.3
	>50	587	34.3
	Missing	74	4.3
**Education level**			
	No formal education	614	35.9
	Primary school	728	42.6
	Secondary school	290	17.0
	Higher education	49	2.9
	Missing	30	1.8
**Residence**			
	Rural	979	57.2
	Semi-urban	729	42.6
	Missing	3	0.2
**Literacy**			
	Literate	1067	62.4
	Illiterate	622	36.4
	Missing	22	1.3
**Agropastoral involvement**			
	Primarily pastoralists	625	36.5
	Primarily peasants	1053	61.5
	Missing	33	1.9
**HIV status**			
	Positive	159	9.3
	Negative	505	29.5
	Test not done	1047	61.2

Of the sputum samples from 1711 study participants who were identified through symptoms and signs of pulmonary tuberculosis, 277 (16%) were confirmed to have mycobacteria by smear microscopy and culture. Of the 1711 samples 228 (13%) were positive by smear microscopy, 123 (7%) by culture and 74 (4%) by both smear microscopy and culture. Among the 123 culture positive, 15 (12.2%) had non-tuberculous mycobacteria. Males were more likely than females to be positive for mycobacteria.

### Association between determinants and mycobacterial disease

**Fig 2:**
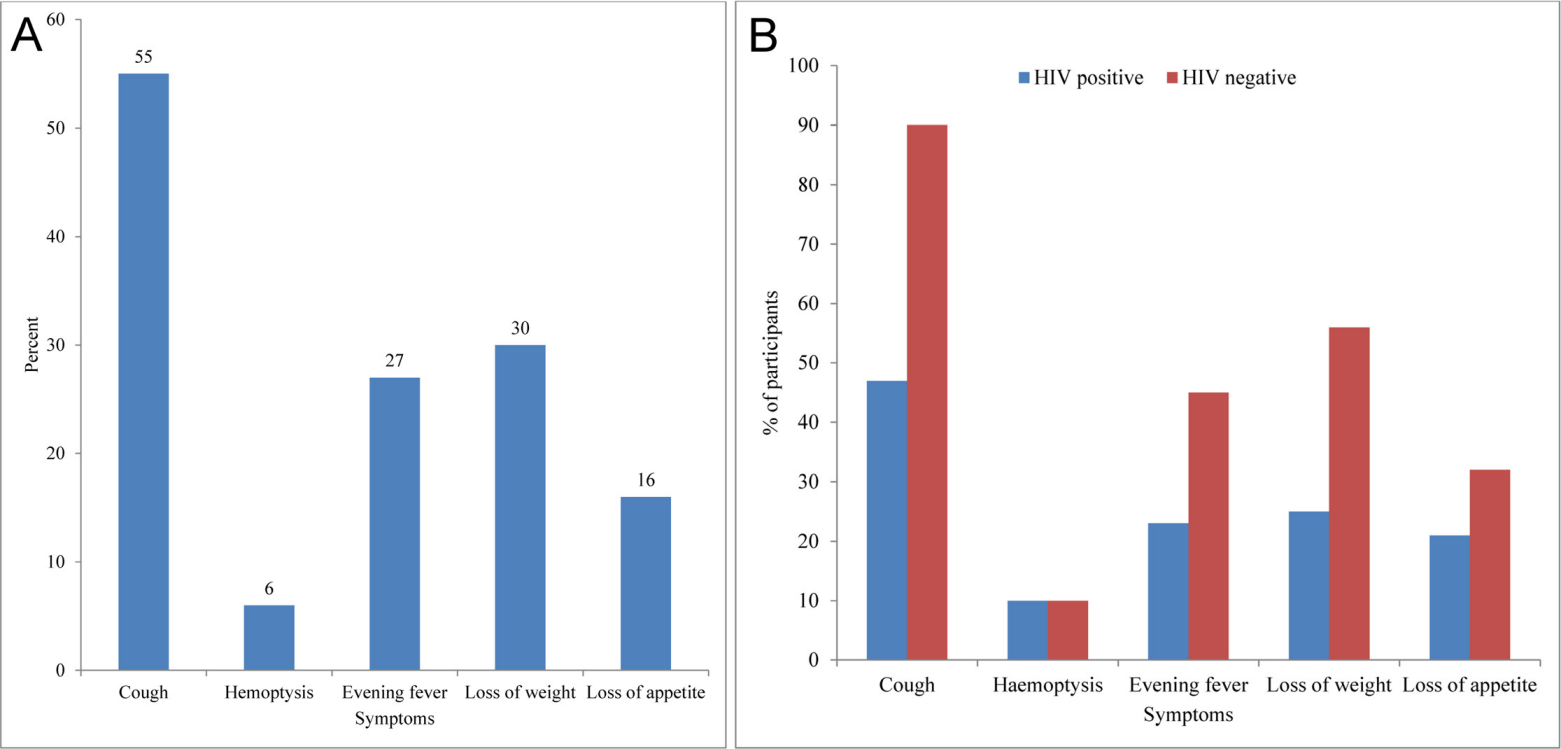
a) Proportion of patients with symptoms for investigation of mycobacterial diseases among 1711 patients attending three tuberculosis clinics in Northern Tanzania, 2010 12. b) Reported symptoms by 664 patients investigated for mycobacterial diseases by HIV status in Northern Tanzania, 2010–12.

In **Table 2**, we show the association between the assessed potential determinants and mycobacterial disease among the study participants. We found higher risk of mycobacterial disease among men, and higher risk among those 40 years or younger compared to those over 50. Loss of appetite was the only symptom significantly associated with being mycobacterial positive among study participants. Of all the study participants, 935 (55%) presented with a persistent cough lasting for two or more weeks (**Fig 2A**), 508 (30%) with loss of weight, 468 (27%) evening fever and 17 (6%) hemoptysis. In **Fig 2B**, we show a comparison of the proportion of reported symptoms by their HIV status.

**Table 2:** Determinants of mycobacterial diseases among 1711 patients examined for tuberculosis in three hospitals of Northern Tanzania, 2010–12.

Determinant		Total Suspects	Mycobacteria (+)	OR (95%CI)	AOR* (95%CI)
		n	n (%)		
Total		1711	277 (16.2)		
**Demographic characteristics**					
Sex					
	Male	927	171 (18.4)	**1.5 (1.1–1.9)**	**1.5 (1.1–2.0)**
	Female	776	104 (13.4)	REF	
	Missing	8	2 (25.0)		
Age group					
	≤20	148	29 (19.6)	1.7 (1.0–2.7)	1.6 (1.0–2.9)
	21–30	276	55 (19.9)	1.7 (1.2–2.5)	1.9 (1.2–2.9)
	31–40	365	73 (20.0)	1.7 (1.2–2.4)	1.7 (1.2–2.6)
	41–50	261	36 (13.8)	1.1 (0.7–1.7)	1.0 (0.6–1.6)
	>50	587	75 (12.8)	REF	REF
	Missing	74	9 (12.2)		
Level of education					
	No formal education	728	123 (16.9)	1.3 (1.0–1.7)	0.9 (0.6–1.4)
	Primary School	290	61 (21.0)	1.7 (1.2–2.4)	1.1 (0.7–1.9)
	Secondary School	49	6 (12.2)	0.9 (0.4–2.1)	0.5 (0.2–1.4)
	Higher Education	615	84 (13.7)	REF	
	Missing	29	3 (10.3)		
Residence					
	Semi-urban	978	159 (16.3)	REF	
	Rural	730	118 (16.2)	1.0 (0.8–1.3)	
	Missing	3	0 (0.0)		
Education status					
	Literate	1067	190 (17.8)	REF	
	Illiterate	623	84 (13.5)	0.7 (0.5–1.0)	
	Missing	21	3 (14.3)		
Agropastoral involvement					
	Primarily peasants	1053	186 (17.7)	REF	
	Primarily pastoralists	626	87 (13.9)	0.8 (0.6–1.0)	
	Missing	32	4 (12.5)		
**Environmental factors**					
Family size					
	6 or less	562	101(18.0)	0.8 (0.6–1.1)	0.9 (0.6–1.2)
	More than 6	1149	177 (15.4)	REF	
Contact with person with tuberculosis					
	Yes	235	37 (15.7)	1.0 (0.7–1.4)	
	No	1413	230 (16.3)	REF	
	Missing	64	10 (15.6)		
Shared a room with domestic animals					
	Yes	564	76 (13.5)	0.8 (0.6–1.0)	1.1 (0.5–2.2)
	No	1136	198 (17.4)	REF	
	Missing	11	3 (27.3)		
Shared water source with animals					
	Yes	589	80 (13.6)	0.7 (0.6–1.0)	0.7 (0.3–1.5)
	No	1114	195 (17.5)	REF	
	Missing	8	2 (25.0)		
Presence of family member with cough					
	Yes	353	53 (15.0)	0.9 (0.7–1.3)	
	No	1328	218 (16.4)	REF	
	Missing	30	6 (20.0)		
Smoking					
	Yes	433	66 (15.2)	0.9 (0.7–1.3)	
	No	1257	205 (16.3)	REF	
	Missing	21	6 (28.6)		
Keeping animals					
	Yes	625	86 (13.8)	0.8 (0.6–1.0)	0.8 (0.4–1.7)
	No	1053	186 (17.7)	REF	
	Missing	33	5 (15.2)		
Previously treated for tuberculosis					
	Yes	92	10 (10.9)	0.7 (0.4–1.3)	
	No	1605	263 (16.4)	REF	
	Missing	15	4 (26.7)		
**Symptoms**					
Cough					
	Yes	935	147(15.8)	0.9 (0.7–1.2)	
	No	771	128(16.6)	REF	
	Missing	5	2 (40.0)		
Hemoptysis					
	Yes	107	17 (16.0)	1.0 (0.6–1.7)	0.9 (0.5–1.9)
	No	1598	257 (16.1)	REF	
	Missing	6	3 (33.3)		
Evening fever					
	Yes	468	75 (16.1)	1.0 (0.8–1.3)	1.0 (0.7–1.6)
	No	1230	199 (16.2)	REF	
	Missing	13	3 (18.8)		
Loss of weight					
	Yes	508	80 (15.8)	1.0 (0.7–1.3)	
	No	1191	193 (16.2)	REF	
	Missing	12	4 (26.7)		
Loss of appetite					
	Yes	285	62 (21.8)	1.6 (1.2–2.2)	2.1 (1.4–3.2)
	No	1414	211 (15.0)	REF	
	Missing	12	4 (26.7)		

*Adjustment factors included: Sex, Age, Education, Residence, Agropastoralist, Coughing family member, and Smoking.

### Association between determinants of and mycobacterial disease among study participants with known HIV status

In **Table 3**, we present an assessment of the association between mycobacterial diseases and its determinants among the HIV positive participants: men were more likely than women to be positive for mycobacteria, and the presence of a family member with a persistent cough also predicted being positive for mycobacteria. In **Table 4** we also show that among the HIV negative participants we found a higher risk of tuberculosis among men than women, among young (≤20 years) patients compared with adults over 50 years, and among those who presented with loss of appetite as a symptom for their illness.

**Table 3:** Determinants of mycobacterial disease among 159 HIV positive patients examined for tuberculosis in northern Tanzania, 2010–12.

Determinant		Total Suspects	Mycobacteria (+)	OR (95%CI)	AOR* (95%CI)
		n	n (%)		
Total		159	21 (13.2)		
**Demographic characteristics**					
Sex					
	Male	78	15 (19.2)	3.0 (1.1–8.1)	2.8 (1.0–8.0)
	Female	81	6 (7.4)	REF	REF
Age group					
	≤20	19	-	**	
	21–30	27	3(11.1)	1.1 (0.2–7.5)	
	31–40	54	12(22.2)	2.6 (0.5–12.7)	
	41–50	33	4(12.1)	1.2 (0.2–7.5)	
	>50	20	2(10.0)	REF	
	Missing	6	-		
Level of education					
	No formal education	104	13(12.5)	**	
	Primary School	26	5(19.2)	**	
	Secondary School	3	0 (0.0)	**	
	Higher Education	25	3(12.0)	REF	
Residence					
	Semi-urban	119	14(11.8)	REF	
	Rural	40	7(17.5)	1.6 (0.6–4.3)	
Education status					
	Literate	133	18(13.5)	REF	
	Illiterate	26	3(11.5)	0.8 (0.2–3.1)	
Agropastoral involvement					
	Primarily peasants	135	17(12.6)	REF	
	Primarily pastoralists	22	4(18.2)	1.5 (0.5–5.1)	
	Missing	2	-		
**Environmental factors**					
Family size					
	6 or less	63	8(12.7)	REF	
	More than 6	96	57(15.4)	0.9 (0.3–3.0)	
Contact with person with tuberculosis					
	Yes	2	0 (0.0)	**	
	No	151	19(12.6)	REF	
	Missing	6	2(33.3)		
Shared a room with domestic animals					
	Yes	23	3(13.0)	1.0 (0.3–3.7)	
	No	136	18(13.2)	REF	
Shared water source with animals					
	Yes	19	3(15.8)	1.0 (0.3–3.9)	
	No	119	18(15.1)	REF	
Presence of family member with cough					
	Yes	3	2(66.7)	15.0 (1.3–173.9)	11.05 (1.1–175.3)
	No	153	18(11.8)	REF	
	Missing	3	1(33.3)		
Smoking					
	Yes	14	3(21.4)	2.0 (0.5–8.0)	
	No	143	17(11.9)	REF	
	Missing	2	1(50.0)		
Keeping animals					
	Yes	22	4(18.2)	1.5 (0.5–5.1)	
	No	135	17(12.6)	REF	
	Missing	2	0 (0.0)		
Previously treated for tuberculosis					
	Yes	9	-	**	
	No	149	21 (14.1)	REF	
	Missing	1	1 (100)		
**Symptoms**					
Cough					
	Yes	65	10 (15.4)	1.4 (0.6–3.5)	
	No	94	11 (11.7)		
	Missing	-	-		
Hemoptysis					
	Yes	13	3 (23.1)	2.1 (0.5–8.5)	
	No	146	18 (12.3)		
	Missing	-	-		
Evening fever					
	Yes	31	6 (19.4)	1.8 (0.6–5.1)	
	No	128	15 (11.7)		
	Missing	-	-		
Loss of weight					
	Yes	34	6 (17.6)	1.6 (0.6–4.4)	
	No	125	15 (12.0)	REF	
	Missing	-	-		
Loss of appetite					
	Yes	27	6 (22.2)	2.2 (0.8–6.4)	1.33 (0.3–5.2)
	No	132	15 (11.4)	REF	

*Adjustment factors included: Sex, Age, Education, Residence, Agropastoralist, Coughing family member, and Smoking.

**Some cells had expected values < 5 making the analysis invalid.

**Table 4:** Determinants of mycobacterial disease among 505 HIV negative patients examined for tuberculosis in Northern Tanzania, 2010–12.

Determinant		Total Suspects	Mycobacteria (+)	OR (95%CI)	AOR* (95%CI)
		n	n (%)		
Total		505	82 (16.2)		
**Demographic characteristics**					
Sex					
	Male	264	49 (18.6)	1.4 (0.9–2.3)	**2.2 (1.3–3.8)**
	Female	239	33 (13.8)	REF	
	Missing	2	-		
Age group					
	≤20	41	11 (26.8)	**2.5 (1.1–5.5)**	**2.5 (1.0–6.3)**
	21–30	78	12 (15.4)	1.2 (0.6–2.6)	1.2 (0.6–2.8)
	31–40	79	18 (22.8)	2.0 (1.0–3.9)	1.8 (0.8–3.7)
	41–50	59	10 (16.9)	1.4 (0.6–3.0)	1.8 (0.6–3.2)
	>50	218	28 (12.8)	REF	
	Missing	30	3 (10.0)		
Level of education					
	No formal education	155	32 (20.6)	1.6 (1.0–2.7)	
	Primary	58	10 (17.2)	1.3 (0.6–2.7)	
	Secondary	7	1 (14.3)	1.0 (0.1–8.7)	
	Higher	277	39 (14.1)	REF	
	Missing	8	-		
Residence					
	Semi-urban	173	32(18.5)	REF	
	Rural	332	50(15.1)	0.8 (0.5–1.3)	
Education status					
	Literate	220	43(19.5)	REF	
	Illiterate	279	39(14.0)	0.7 (0.4–1.1)	
	Missing	6	-		
Agropastoral involvement					
	Primarily peasants	166	36(21.7)	REF	
	Primarily pastoralists	338	46(13.6)	0.6 (0.4–0.9)	
	Missing	1	-		
**Environmental factors**					
Family size					
	6 or less	135	25(18.5)	REF	
	More than 6	370	57(15.4)	0.8 (0.4–1.5)	
Contact with person with tuberculosis					
	Yes	130	22(16.9)	1.1 (0.6–1.8)	**2.1 (1.0–4.5)**
	No	365	59(16.2)	REF	
	Missing	10	1(10.0)		
Shared a room with domestic animals					
	Yes	313	43 (13.7)	0.6 (0.4–0.1)	1.4 (0.4–5.0)
	No	191	39 (20.4)	REF	
	Missing	1	-		
Shared water source with animals					
	Yes	306	41 (13.4)	0.6 (0.4–1.0)	0.6 (0.2–2.0)
	No	199	41 (20.6)	REF	
Presence of family member with cough					
	Yes	204	28 (13.7)	0.7 (0.4–1.2)	0.7 (0.3–1.7)
	No	296	54 (18.2)	REF	
	Missing	5	-		
Smoking					
	Yes	224	30 (13.4)	0.7 (0.4–112)	0.9 (0.5–1.8)
	No	277	51 (18.4)	REF	
	Missing	4	1 (25.0)		
Keeping animals					
	Yes	338	46 (13.6)	0.6 (0.4–0.9)	0.5 (0.2–1.6)
	No	166	36 (21.7)	REF	
	Missing	1	-		
Previously treated for tuberculosis					
	Yes	28	3 (10.7)	0.6 (0.2–2.0)	
	No	474	79 (16.7)	REF	
	Missing	2	-		
**Symptoms**					
Cough					
	Yes	395	59 (14.9)	0.7 (0.4–1.1)	
	No	110	23 (20.9)	REF	
	Missing	-	-		
Hemoptysis					
	Yes	45	6 (13.3)	0.8 (0.3–1.9)	
	No	459	76 (16.6)	REF	
	Missing	1	-		
Evening fever					
	Yes	201	27 (13.4)	0.7 (0.4–1.2)	0.7 (0.4–1.3)
	No	304	55 (18.1)	REF	
	Missing	-	-		
Loss of weight					
	Yes	245	38 (15.5)	0.9 (0.6–1.4)	
	No	258	44 (17.1)	REF	
	Missing	2	-		
Loss of appetite					
	Yes	131	29 (22.1)	**1.7 (1.0–2.8)**	**2.8 (1.5–5.2)**
	No	373	53 (14.2)	REF	
	Missing	1	-		

* Adjustment factors included in the final model were: Sex, Age, Education, Residence, Agropastoralist, Coughing family member, and Smoking.

## Discussion

The current study shows that pulmonary mycobacterial diseases were common among the investigated patients. Men had higher risk of mycobacterial diseases both among HIV positives and HIV negatives, as well as the HIV positive patients who had a family member with persistent cough. Young adults and patients presenting with loss of appetite also were at increased risk of mycobacterial disease.

We demonstrate that non-tuberculous mycobacteria were prevalent among the participants examined in the agropastoral communities in northern Tanzania. Since patients with non-tuberculous mycobacteria present with acute or chronic illness that is clinically and radiologically indistinguishable from *M*. *tuberculosis*, misdiagnosis of non-tuberculous mycobacteria infection could therefore lead to inappropriate anti-tuberculosis treatment. In Tanzania, the major diagnostic method for tuberculosis is sputum smear microscopy with culture only done at the CTRL and some few zonal laboratories. For that case non-tuberculous mycobacteria cases with positive smears will continue to be misclassified as *M*. *tuberculosis* and subsequently treated with conventional anti-tuberculosis drugs to which some of them may be resistant and a large majority of non-tuberculous mycobacterial infections will remain undetected.

### Occurrence of mycobacterial diseases in the study area

Information on the prevalence of diseases such as tuberculosis is vital for planning, implementation and evaluation of control strategies at local, national and global levels. In the current study, we report that the majority of the patients found to have mycobacteria had a positive sputum smear. We think part of the reason that many acid fast bacilli positives were negative on culture may have been due to long transport time. Still the proportion of “presumptive tuberculosis patients” who were finally reported to be mycobacterial positive (the yield) was higher than the national and regional smear positive tuberculosis rates [18]. Reports from other African countries have also documented varying prevalence of mycobacteria, indicating their public health importance in agropastoral communities [19,20]. Factors such as HIV, patient’s understanding about the disease, and an increased role of environmental sources and livestock/wildlife reservoirs have been reported to play role in the existence of mycobacterial diseases in humans [2,21,22].

### Association between demographic determinants and mycobacterial diseases

Diagnosis of pulmonary tuberculosis based on a combination of clinical symptoms, sputum microscopy for acid-fast bacilli and chest radiography have been reported to be fairly sensitive, but nonspecific [23]. A study conducted earlier reported that age and symptoms were useful in predicting and screening for smear-negative pulmonary tuberculosis suspects and cases [24]. Predictors of mycobacterial diseases reported in this study were in line with those reported in a study conducted to evaluate the clinical, diagnostic and epidemiological characteristics of patients suspected to have pulmonary tuberculosis in Ethiopia [23]. This observation indicates that if used as a tool to support the diagnosis of mycobacterial diseases, clinical symptoms are useful, although their use may require some caution. The general rule in diagnosing mycobacterial diseases, including pulmonary tuberculosis involves examination of a patient with a cough or expectoration for two or more weeks by smear microscopy or chest radiograph [25]. In addition, in order to find patients with mycobacterial diseases, clinicians inquire about symptoms, risky exposures and habits that may suggest the need for further investigation [24]. In the current study, we found that sex, age and loss of appetite were associated with being mycobacteria positive, regardless of the HIV sero-status of the individual. Our findings align well with studies conducted in other developing countries [26–29].

### Association between potential determinants and mycobacterial diseases by HIV status

Among the mycobacterial diseases associated with HIV infection, tuberculosis is of particular importance [30]. People infected with HIV have ten times higher risk of developing tuberculosis than healthy people, and pulmonary tuberculosis is still the most common form [31]. Co-infection with HIV has a major effect on the natural history of many infectious diseases, particularly mycobacterial diseases [32]. HIV infection has been reported to affect the diagnosis of pulmonary tuberculosis in HIV positive patients [33]. In our study, we found that among HIV positive individuals, having a family member with a persistent cough was associated with being mycobacteria positive. It is well known that persons in the household of a tuberculosis patient are exposed to the bacteria and may develop disease, but for clinical practice, this is not among the “classic” risk factors for identifying patients to be examined for tuberculosis. In our study, this was not a significant risk factor among HIV negative patients, but for HIV positive patients, it was a very strong risk factor. Although there is no conclusive evidence that HIV sero-positive persons are more likely to acquire tuberculosis infection than HIV sero-negative individuals given the same degree of exposure [34], the risk of rapid progression is much greater among persons with HIV infection, as HIV impairs the host's ability to contain new tuberculosis infection. HIV co-infection also increases the risk of progression of recently acquired infection to active disease [34,35]. The impact of HIV on the epidemiology, natural history, and clinical presentation of mycobacterial diseases, especially tuberculosis, has been well documented in previous studies, and it may explain the reported findings [36,37]. The reported and observed limited clinical symptoms show that when diagnosing mycobacterial diseases in persons with known or possible HIV infection, one has to consider using an appropriate diagnostic and screening approach. Although screening for mycobacterial diseases using symptoms does not require expensive equipment or specialized health personnel, the sensitivity and specificity of symptoms as a tool for diagnosis of tuberculosis has been reported to be lower in immune suppressed HIV individuals [33].

This study shows that for HIV negative individuals who had a family member who had been coughing for two or more weeks, being a male and aged 20 years or younger were significantly associated with being mycobacteria positive. Other studies show that screening by cough alone in HIV positive patients has low sensitivity [38**–**42], with up to 86% of tuberculosis cases being missed. In a study conducted in Cambodia, it was reported that the sensitivity of using symptoms rose when fever and weight loss were included as symptoms of mycobacterial disease [40]. This study has some limitations. Assessment of the HIV status of the suspected tuberculosis patients involved in this study relied on patient records available at the health facilities. As part of the national policy, all suspected tuberculosis patients are tested for HIV. However, due to poor record keeping and logistical issues, we found that more than half of the tuberculosis patients lacked HIV test results in the health facility register. This resulted to a lower strength of our associations, and care must be taken in interpretation when no associations between determinant and mycobacterial disease are found. Also, in the selection of all participants by symptoms, even the comparison group represents “suspects”, not healthy individuals. That means we already selected those with symptoms, so we cannot really say how well symptoms predict mycobacterial disease such as tuberculosis in the population, only how well it predicts disease among suspects. This will confound and weaken the associations between symptoms and disease, and in our study many symptoms will not predict disease in the normal population. Furthermore, many variables (e.g. symptoms) depend on participants’ recall and understanding about the disease and thus do not always represent the objective reality. However, this parallels the situation for clinicians, who are often more dependent on recall than objective reality.

## Conclusion

The findings in this study show that both *M*. *tuberculosis* and non-tuberculous mycobacterial strains were prevalent in the study community. The high proportion of nontuberculous mycobacteria among the participants indicates clinical and environmental occurrence and possible human-environment-livestock risks of cross transmission. Some risk factors were identified which may improve screening for mycobacterial diseases, but their use requires some precaution.
